# MaxEnt model-based prediction of potential distributions of *Parnassiawightiana* (Celastraceae) in China

**DOI:** 10.3897/BDJ.10.e81073

**Published:** 2022-03-16

**Authors:** Xiaosong Dai, Wei Wu, Ling Ji, Shuang Tian, Bo Yang, Bicai Guan, Ding Wu

**Affiliations:** 1 College of Life Science, Nanchang University, Nanchang, China College of Life Science, Nanchang University Nanchang China; 2 The Institute for Sustainable Development, Macau University of Science and Technology, Macau, China The Institute for Sustainable Development, Macau University of Science and Technology Macau China; 3 Jiangxi Agricultural University, Nanchang, China Jiangxi Agricultural University Nanchang China; 4 Jiangxi Key Laboratory of Plant Resources and Biodiversity, Jingdezhen University, Jingdezhen, China Jiangxi Key Laboratory of Plant Resources and Biodiversity, Jingdezhen University Jingdezhen China

**Keywords:** *Parnassiawightiana*, bioclimatic variables, MaxEnt, potential suitable region

## Abstract

The maximum entropy (MaxEnt) model for predicting the potential suitable habitat of species has been commonly employed in many ecological and biological applications by using presence-only occurrence records along with associated environmental factors. *Parnassiawightiana*, a perennial herb, is a cold-adapted plant distributed across three diversity hotspots in China, including the Hengduan Range, Central China and the Lingnan region. The MaxEnt model was used to simulate the historic, current and future distribution trends of *P.wightiana*, as well as to analyse its distribution pattern in each historical period and explore the causes of species distribution changes. The results of our analysis indicated that annual precipitation, annual temperature range and mean temperature of the warmest quarter were the key bioclimatic variables affecting the distribution of *P.wightiana*. Most temperate species retracted into smaller refugial areas during glacial periods and experienced range expansion during interglacial periods. Possible refugia of the species were inferred to be located in the Hengduan Range and Qinling Regions.

## Introduction

Climatic oscillations during the Cenozoic period, especially the Quaternary glacial/interglacial cycle, had a great impact on the geographical distribution patterns and genetic structure of species (Hewitt 1999, Hewitt 2004). As such, the impact of climate change on species distributions has always been a popular issue in biogeographic research (Fitzpatrick et al. 2008). In general, most temperate species retracted into smaller refugial areas during glacial periods, followed by range expansion during interglacial periods (Hewitt 1996, Hewitt 2000, Bueno et al. 2016). Nevertheless, continued global warming in the future may pose a serious threat to cold-adapted species, especially those currently confined to mountaintops or islands, because migration to higher elevations may be not possible or range shifts are not fast enough to track suitable climate (Wiens 2016). Therefore, it is of great theoretical and practical importance to understand potential climatic suitability areas during different periods, as well as the dynamics of species populations in order to protect biodiversity under future climatic conditions.

*Parnassiawightiana* Wall. ex Wight & Arn. is an ancient plant species distributed over the China-Himalayan Region (Wu 2003). The geographic distribution of the species encompasses three biodiversity hotspots with high richness and endemism defined by Ying Junsheng, namely the Hengduan Range, Central China and the Lingnan Region in China (Ying 2001). *Parnassia* L. is one of the genera of the family Celastraceae, according to APG IV, comprising about 60 species globally (Shu 2017). The Himalaya-Hengduan Mountains represent the distribution and differentiation centre of the genus (Wu 2003). *Parnassiawightiana*, commonly known as Chicken-leaf Grass, is a perennial herb and adapted to both temperate and cold environments (Ku 1987, Wu 2005). Suitable habitat for *P.wightiana* typically includes alpine meadows, forest edges and stream banks, indicating a history of glacial range expansion and postglacial contraction (Wu 2003, Wu 2005). Recently, species distribution models (SDM) have been applied to infer range shifts of plants and animals in response to the Quaternary climate oscillations (Elith and Leathwick 2009, Luo et al. 2016). Herbaceous plants are capable of far more life cycles within a given time period and, thus, may respond more quickly to rapidly changing environments compared to slower regenerating organisms (Comes and Kadereit 1998, Niu et al. 2017).

Species distribution models are an approach that identifies and describes potential suitable habitat for species (Elith and Leathwick 2009). MaxEnt (Maximum entropy models, https://biodiversityinformatics.amnh.org/open_source/maxent/) are one of the most popular tools for modelling species distributions. Specifically, presence-only occurrence records and relevant environmental variables may be used to build a final habitat map to obtain the best estimate of the species distribution (Phillips et al. 2006). To date, SDMs have been widely applied to areas such as the prediction of potential distribution of species, the protection of rare plants and animals, the prevention of invasive organisms and paleontological geography (Xu et al. 2015, Cao et al. 2020, Chen et al. 2021, Lompo et al. 2021, Tang et al. 2021, Wang et al. 2021).

At present, the research on *P.wightiana* only focuses on the development of taxonomy and medicinal value and there is no report on the change of distribution pattern in different periods under the background of climate change. The field survey found that the wild population size of *P.wightiana* is becoming smaller and smaller and, due to climate change and human disturbance, the wild living environment has been very bad. There is an urgent need to pay attention to the protection of wild *P.wightiana* population and study its distribution pattern with climate change. In this study, the cold-adapted species *P.wightiana* served as the focal species. A MaxEnt model was then used to reconstruct the potential species distribution in five periods, namely the Last Interglacial (LIG), Last Glacial Maximum (LGM), Mid-Holocene (MH), Current (1950-2000s) and Future (2070s) and the simulation results were calculated and visualised by ArcGIS 10.4 (ESRI, Redlands, CA, United States; www.esri.com) (Hernandez et al. 2006, Phillips et al. 2017). The aims of this study were to: (1) obtain the change rule of the distribution pattern of *P.wightiana* with the change of environment；(2) according to the Jackknife method and environmental variables assessment, to understand the main environmental factors affecting its distribution and (3) learn about the refuge position of *P.wightiana*. The results can provide a theoretical basis for the future development and utilisation of resources and population ecological protection of *P.wightiana*.

## Materials and methods

### Natural distribution data

Distribution data of *P.wightiana* were sourced from field surveys and the Chinese Virtual Herbarium (CVH, http://www.cvh.ac.cn). Based on the National Natural Science Foundation of China (No.4156010383), 45 natural populations of *P.wightiana* were obtained by extensive field investigation. Additionally, complete distribution information from CVH were first confirmed and then recorded. If there was a detailed collection site, the longitude and latitude were located using Google Earth with reference to the recorded habitat, altitude, along with other information. Voucher photos were checked for records sourced from CVH and NSII (National Specimen Information Infrastructure) to confirm that the species were correctly identified. In total, 48 collection entries from herbarium research were obtained after removing repetitive entries and identification errors.

**Figure 1.**
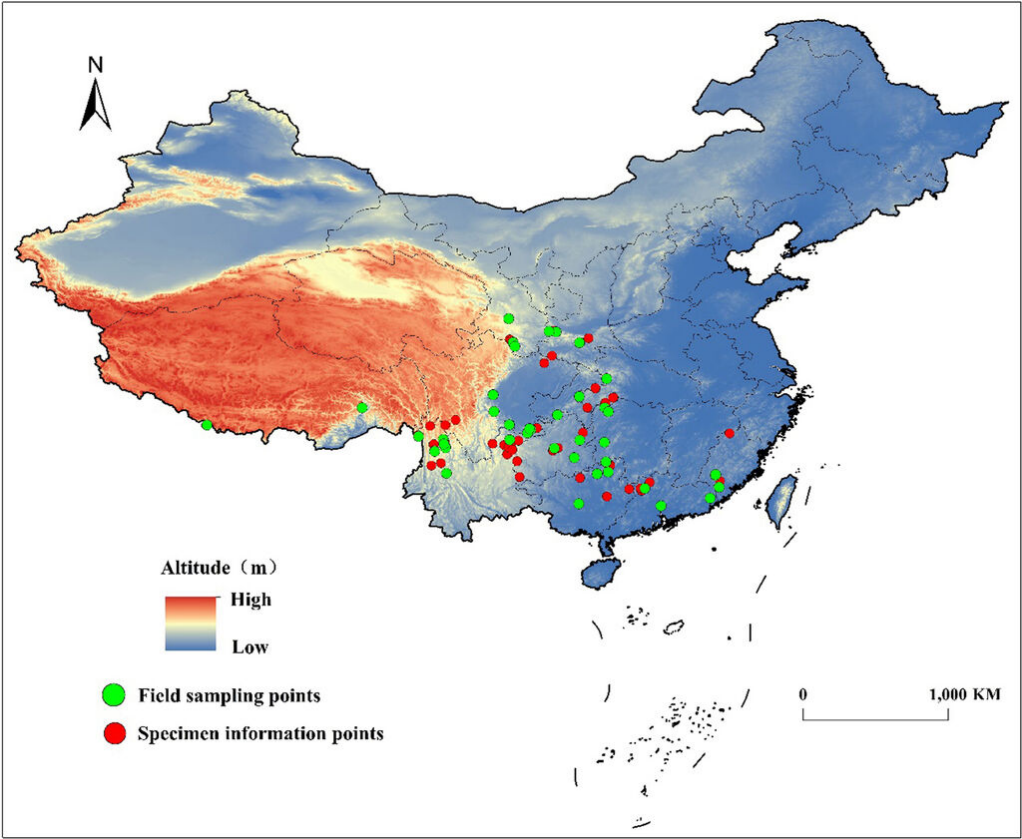
Geographic distribution sample points of *Parnassiawightiana*.

To prevent model over-fitting caused by data repetition and spatial autocorrelation, as well as to associate model error to the results, the buffer method was used to screen the obtained data. The spatial resolution of environmental variables was 2.5 arc-minutes and spatially coincident data points within 3 km of each other were also eliminated (Wang et al. 2017, Guo et al. 2019). Finally, a total of 91 filtered occurrence records were obtained from CHV and NSII, along with our own collections, which well covered the distribution range of *P.wightiana* (Fig. 1, Suppl. material 1)

### Environmental variables

The bioclimatic variables (Hijmans et al. 2005), downloaded from the WorldClim database (http://www.worldclim.org), included nineteen climatic factors representing historical periods, i.e. LIG, LGM, MH, along with Current and Future periods. Future climate selection RCP (Representative Concentration Pathways) 6.0 represents the greenhouse gas emission peak scenario for projections over the distribution area (Wang et al. 2017). The common climate model CCSM4 (Community Climate System Model) was used to source the environmental data for each period and the 2.5 arc-minutes spatial resolution was adopted. The geographical coordinates were unified as GCS_WGS_1984. The ArcGIS 10.4 extract by mask tool was used to cut and extract the environmental variable data, retaining the required range of data and, finally, to convert to ASCII format. A 1:4 million scale provincial administration map was downloaded from the China's National Basic Geographic Information System (http://nfgis.nsdi.gov.cn) as the analysis base map.

High correlation and collinearity between bioclimatic variables can easily lead to the over-fitting of the model, thus affecting the accuracy of the resulting predictions, so SPSS 26 was utilised to perform principal component analysis on all bioclimatic variables (Yang et al. 2013). Bioclimatic variables corresponding to the species distribution points were extracted and then imported into MaxEnt v.3.4.1 together with the species data for suitability prediction, where the climate variables greater than 1% in the model prediction results were retained (Shi et al. 2021). To eliminate multicollinearity effects in the parameter estimates of the SDM, variables with Pearson’s |R| ≥ 0.85 were excluded (Yusup et al. 2018) and only six variables were selected as climatic predictors to model the past, current and future climatically-suitable areas of *P.wightiana*. These six bioclimatic variables, namely Bio2, Bio4, Bio7, Bio11, Bio12 and Bio14, were retained for the construction of the distribution prediction model (Table 1).

### Model construction

The sorted distribution point data and the screened bioclimatic variable data were imported into MaxEnt 3.4.1 and the bioclimatic variables were evaluated by the Jackknife test. The models obtained were calibrated using 75% of the available records for each species as training (calibration) data and the remaining 25% were used for model validation as test data. The Bootstrap method was implemented with 10 repeats, a maximum of 5000 iterations and default selected parameters (Yan et al. 2020).

Model performance was evaluated by calculating the Area Under the Receiver Operator Curve (AUC), where models with AUC values larger than 0.7 were considered satisfactory for our study (Wiley et al. 2003, Phillips and Dudik 2008). MaxEnt is a multivariate approach that estimates the distribution of a species by finding the probability distribution of maximum entropy, subject to constraints representing our incomplete information about the distribution. It is generally understood that AUC < 0.7 indicates low accuracy of the model, prediction results can be adopted when AUC is 0.7–0.9 and AUC > 0.9 indicates that the prediction results are very accurate, which can be used for subsequent analysis.

## Results

### Classification of suitable areas

**Figure 2.**
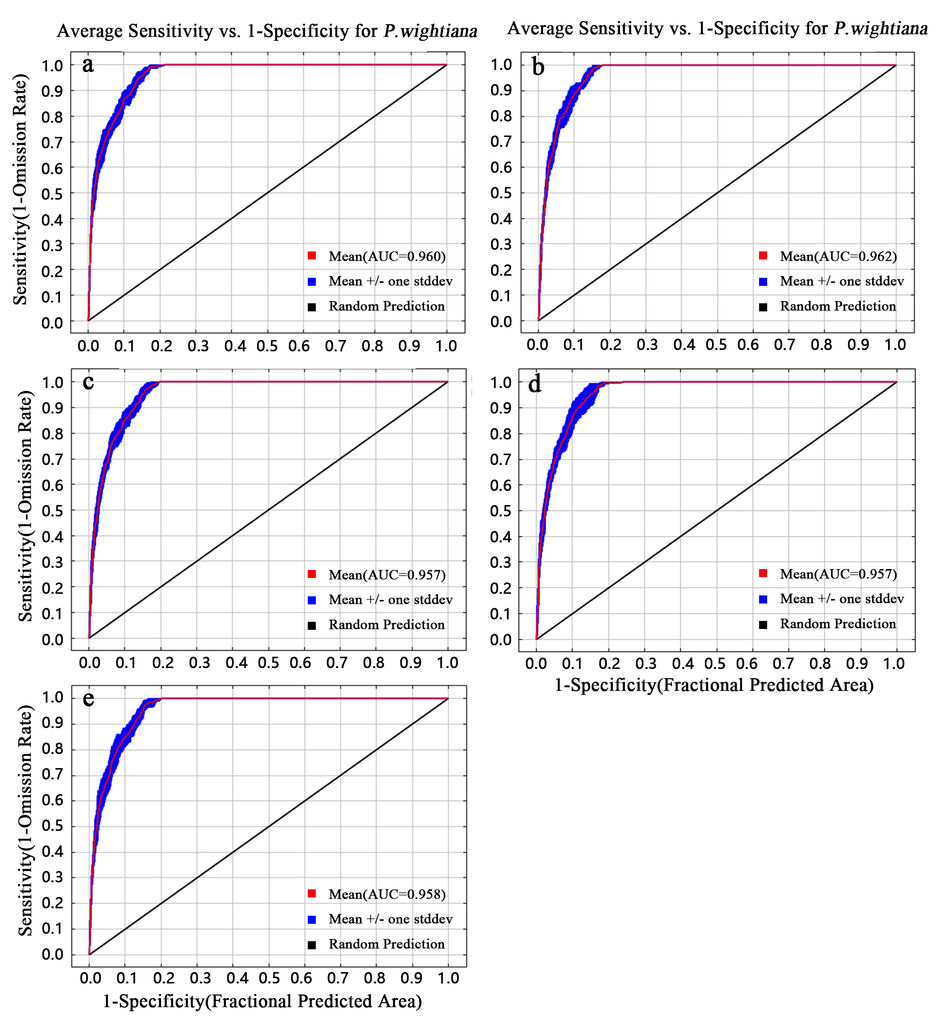
The results of the AUC in developing *Parnassiawightiana* habitat suitability model. **Note**:*a: LIG, b: LGM, c: MH, d: Current, e: Future.

A MaxEnt model was used to predict the distribution of *P.wightiana* in different periods, which were then visualised using ArcGIS. The prediction of the MaxEnt model, based on the current climate data, is very consistent with the actual distribution area and the AUC values of each period are greater than 0.9, indicating that the model has a good predicting ability (Fig. 2).

To distinguish unsuitable habitat from suitable habitat, a reclassification of the probability maps was performed using a threshold, which establishes the minimum level below which a given distribution should be excluded. The threshold of automatic generation, based on the model, is 0.277, that is, the range of 0-0.277 represents the non-suitable area of *P.wightiana*. Suitable areas are divided into the following grades: low suitable areas (0.277-0.518), medium suitable areas (0.518-0.759) and high suitable areas (0.759-1). Total counts of the grid numbers of each grade were used to calculate the area of each suitable region (Table 2).

### Main climatic factors

The most influential environmental variables (Table 1) used by the Maxent model for the best model performance were Bio2, Bio4, Bio7, Bio11, Bio12 and Bio14. The contribution rates of annual precipitation (Bio12), annual average temperature range (Bio7) and mean temperature of the warmest quarter (Bio10) in each period were ranked in the top three and constituted more than 17% of the total contribution. The six bioclimatic variables, selected here, are known to influence the distribution and physiological performance of plant species (Table 3). *Parnassiawightiana* is sensitive to habitat and climatic changes and requires a specific forest habitat that is rich in water and adequate temperature. Results from the Jackknife test indicate that precipitation was the most influential factor.

### Species distribution modelling

#### Distribution area under current climate

**Figure 3.**
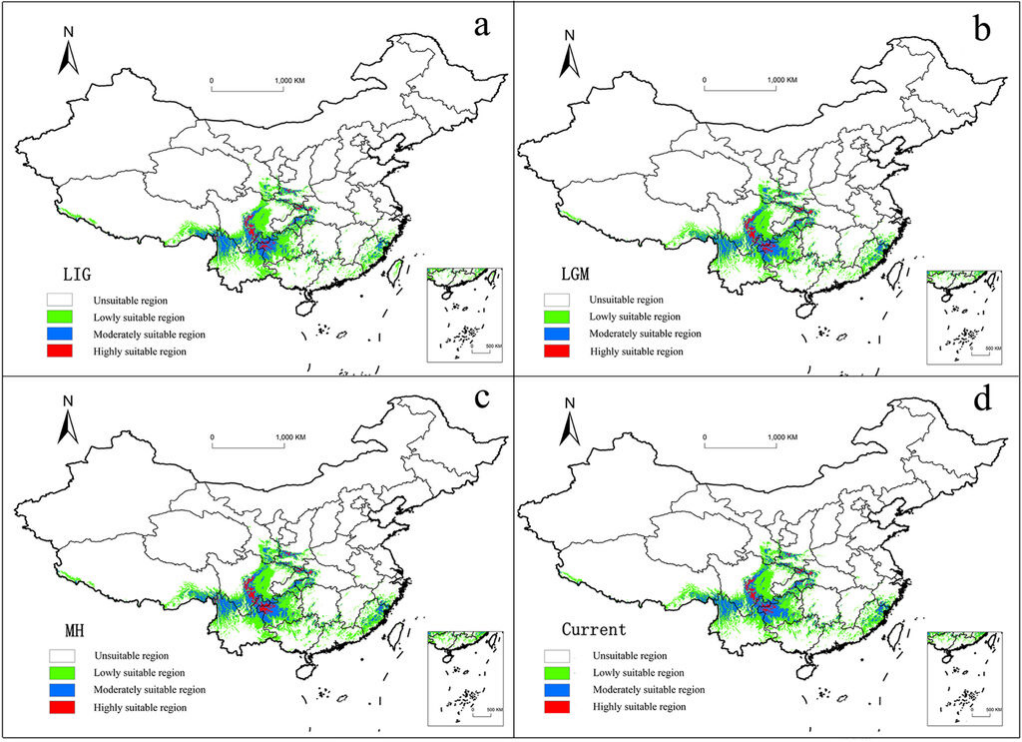
Prediction of the potential distribution of *Parnassiawightiana* over four periods.

An estimate of the current distribution of *P.wightiana* was developed using the same six bioclimatic variables for current climate (1950-2000s). The total predicted suitability area was 85.04×10^4^ km^2^, of which the high suitability area was 3.52×10^4^ km^2^, the medium suitability area was 25.96×10^4^ km^2^ and the low suitability area was 55.56×10^4^ km^2^ (Table 2). Additionally, these results indicate that the moderately/high suitability areas are mainly located in the Hengduan and Qinling Mountains, as well as occasionally in the Lingnan Region (Fig. 3d).

The entire geographical range of *P.wightiana* follows very closely to the area of China's mountains. Sichuan Basin is characterised as a non-suitable area, which is consistent with the modern habitat distribution of *P.wightiana*. Compared to the actual distribution, it is found that the range predicted by the model is in general agreement with the actual distribution area. Although there are minor deviations, the core distribution area is consistent with the current distribution.

#### Prediction of suitable areas in historical periods

Our SDM analysis provides a detailed picture of the last glacial cycle (Fig. 3a，b and c) as well as glimpses of the preceding cycles. During the Last Interglacial period, the geographical distribution of *P.wightiana* exhibits some similarity to that currently observed. The suitability area during this period was mainly distributed around the Sichuan Basin and the overall trend was consistent with the topography of the south-western mountains of China. A total of suitability area was 79.17×10^4^ km^2^, of which 4.45% were highly suitable and concentrated in high altitude areas, such as the Yunnan-Guizhou Plateau along with the Hengduan, Qinling and Daba Mountains (Fig. 3a).

**Figure 4.**
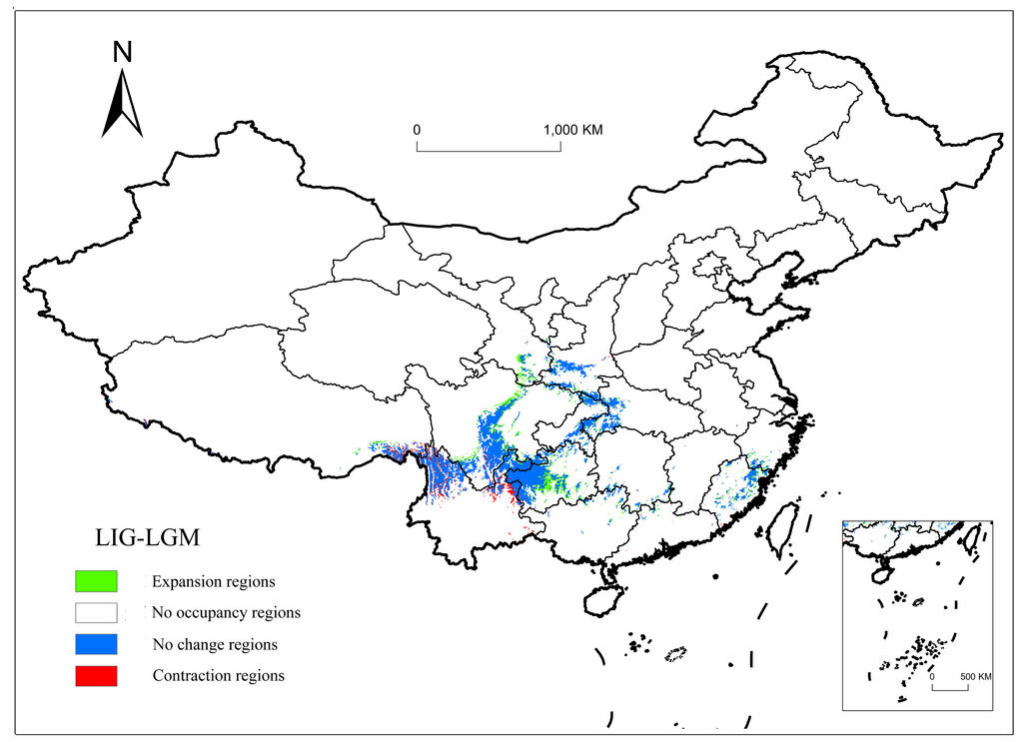
Range shifts of mid-high suitable areas from the LIG to the LGM.

The potential suitable habitat for *P.wightiana* increased slightly during the Last Glacial Maximum, with a total area of 82.08×10^4^ km^2^, while the high climatic suitability area increased from 3.52×10^4^ km^2^ to 4.18×10^4^ km^2^ (Table 2). The climatic characteristics of the glacial period are characterised by dry and cold conditions and, as such, cold-adapted taxa that inhabited high mountains migrated to higher elevations, especially in central Guizhou and northern Sichuan. Central China provides a favourable environment for migration and preservation, while the dry valley of the Hengduan Mountains leads to contraction. Potential refugia, recognised by this study, are encircled by blue areas (Fig. 4).

During the Holocene, as temperatures generally increased, the distribution range for *P.wightiana* shrank and suitable areas at all levels decreased. The high suitability area was 3.45×10^4^ km^2^ and the total area decreased by 4.71×10^4^ km^2^ (Fig. 3c). Additionally, as China did not directly experience the effects of the Quaternary continental glaciers, many places, therefore, served as sheltered habitat for Tertiary flora in tropical and subtropical areas. The geographical range of *P.wightiana* in the Lingnan Region was limited to forest edges and areas near streams and the suitability areas were small and scattered, including areas, such as the Jiulian Mountain in Guangdong and the Lianhua Mountain in Fujian.

#### Future potential distribution

The results from these analyses predicted that the future distribution range of *P.wightiana* will shrink and that the total area may be reduced to 82.89×10^4^ km^2^. The medium and low suitability areas are predicted to be reduced, but the high suitability areas will remain stable. Numerous suitability areas that were previously identified in Sichuan Basin are predicted to no longer be suitable habitat for the survival of *P.wightiana*. Under continued global warming into the future, we observe a migration to higher elevations and the habitat becoming fragmented in Wumeng Mountain of Yunnan-Guizhou Plateau. The medium suitable areas in central Guizhou are predicted to shift to low suitable areas.

**Figure 5.**
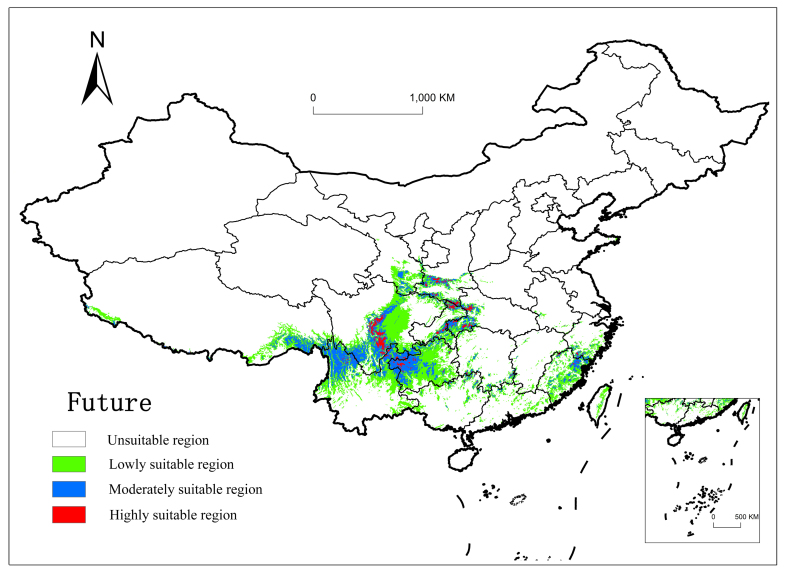
Distribution prediction of *Parnassiawightiana* under future climatic conditions.

The predicted suitable habitats of *P.wightiana* under current conditions were generally in agreement with the actual observed species distribution (Fig. 3d). These findings suggest that, in general, high climatic suitability areas for *P.wightiana* by the 2070s may experience a northward range shift in Mainland China and suitability may slightly decline in the southern Hengduan Mountains region (Fig. 5).

## Discussion

### Influence factors of simulation accuracy

In the present study, we found that the MaxEnt model was able to provide robust results with small, sparse and irregularly sampled data and our results are visualised using ArcGIS. The operation was simple and intuitive and the sample requirement was small and other previous studies have also highlighted the great performance of similar methods. For example, De Siqueira et al. (2009) identified a rare plant species that had already been declared extinct through ecological niche simulation. For many species, in fact, the sample field data are insufficient to characterise the geographic distribution of species in the study area and, often, must be supplemented by digital specimen information. However, the distribution records of some specimens must be confirmed by Google Earth due to the lack of detailed latitude and longitude information. Some distribution sites have been collected over long periods and their environment has changed over time. Using these distribution data for simulation often weakens the model's niche definition, resulting in a decline in the simulation ability and accuracy of the distribution model.

In this study, the environmental variables used in the MaxEnt model are all climate factors. Nineteen climatic variables, based on temperature and rainfall, were chosen according to the different needs of computing performance. However, there are many factors in addition to climate that affect the distribution of species, such as interspecific interactions, microtopography and local microclimates. For example, the uplift of the Qinghai-Tibet Plateau, along with the environmental destruction caused by human activities may together lead to species extinction (Montemayor et al. 2015). Therefore, the MaxEnt model represents the theoretical maximum possible species distribution and the suitable areas are often shown to be much wider than the actual areas occupied. The application of models, based on the principle of maximum entropy for predicting species distributions according to the extent of suitable area, represents a useful tool, both to define the most important environmental variables responsible for current distributions and to provide useful information for their conservation.

### Distribution pattern and potential refugia

The predicted distribution of *P.wightiana* in each period showed that the suitable areas were concentrated in the mountainous areas of south-western China, mainly in the Himalayan, Hengduan and Qinling Mountains. According to the existing collection along with specimen records, Daming Mountain in Wuming District in Guangxi represents the southern distributional limit, the Weiyuan Weihe River in Gansu represents the northern, Huanggang Mountain in Jiangxi the eastward and Jipu Village in Jilong Town in Tibet constitutes the westward limit. Additionally, Nepal, Bhutan, northern Myanmar, Thailand, Sikkim and northeast India are also distribution areas for *P.wightiana* (Shu 2017). Overall, the distribution range (23°-35°N, 85°-118°E) spans over three endemic centres, one in the Hengduan Mountains, another in Central China and finally in the Lingnan Region of China, showing an obvious discontinuous distribution.

Based on the results of the MaxEnt model, the key factors affecting species geographical distribution are not completely consistent. For example, a previous study on *Stipapurpurea* in the Tibetan Plateau found that precipitation was a key factor affecting its distribution (Hu et al. 2015). For *Rosaroxburghii*, temperature displayed the greatest influence on the future distribution in a simulation study (Fan et al. 2021). The results of our analysis indicated that annual precipitation was the most important climatic factor affecting the distribution of *P.wightiana*. The species is mainly distributed along the edge of the Sichuan Basin and is confined to mountaintops. Therefore, average annual temperature and the average temperature in the warmest season are the key factors affecting the distribution of *P.wightiana*.

Understanding species distributional patterns is a fundamental question in biogeography and conservation biology. The middle and high suitable areas of *P.wightiana* were mainly located in the Hengduan Mountains, Yunnan-Guizhou Plateau, Qinling Mountains and Daba Mountain at high altitudes. During the Last Glacial Maximum, as temperatures gradually began to decline, some inland areas of Sichuan Basin began to transform into suitable areas. The Weihe River source in Gansu Province was a residual area of the Qinling Mountains and also began to transform into a medium suitable area. For the cold-adapted *P.wightiana*, the decline in temperature makes the environment more suitable for survival, most likely during the glacial expansion and postglacial contraction.

The study of potential plant refuges during the Quaternary glaciation is of great significance for understanding current plant distribution patterns along with future evolution. A MaxEnt model was employed to simulate the distribution of highly suitable areas over different time periods, allowing for the inference of potential refugia (Chan et al. 2011, Bai and Zhang 2014). The results of our analysis suggest that the Hengduan Mountains, Yunnan-Guizhou Plateau and Qinling Mountains represent the core areas of the potential distribution of *P.wightiana*, which provide more suitable habitats for survival than other areas. These findings may, therefore, provide useful information for increased understanding of the origin and differentiation of northern temperate species.

### Suggestion on wild population protection of Parnassiawightiana

In this study, the distribution range of *P.wightiana* in the future was predicted. The results showed that the suitable area of *P.wightiana* would continue to shrink in the next few decades. Although the highly suitable area remained stable, the prospect of population development was not optimistic. In view of the small size of wild populations and serious human disturbance, we put forward some suggestions: (1) in situ conservation of the known *P.wightiana* community, minimise the adverse effects of human activities on species survival; (2) at present, the research on biological characteristics, artificial cultivation methods and genetic shape improvement of *P.wightiana* is relatively weak and systematic research in this area should be strengthened; (3) artificial harvesting and planting can be carried out around some populations with less human activity to maximise the size of their wild populations.

## Conclusion

The simulation results show that the current suitable area is basically consistent with the actual distribution area and the AUC values in each period are greater than 0.9, indicating that the results are accurate. In the change of distribution pattern, the *P.wightiana* accorded with the pattern of glacial expansion and interglacial contraction, but the overall change of area and scope was not obvious. The main environmental factors affecting the distribution of *P.wightiana* are annual precipitation (Bio12), annual temperature range (Bio7) and average temperature in the warmest quarter (Bio10). *P.wightiana* may have multiple refuges, distributed in Hengduan Mountains, Qinling Mountains and other high mountains. The future climate is not ideal for the survival of *P.wightiana* and local protection and artificial breeding are important ways to protect wild populations of *P.wightiana*.

## Supplementary Material

DA785FC6-615A-5148-85E5-ABCC64AA155F 10.3897/BDJ.10.e81073.suppl1 Supplementary material 1 Species distribution information Data type Table File: oo_634132.xlsx https://binary.pensoft.net/file/634132 Ding Wu

## Figures and Tables

**Table 1.** Description of bioclimatic variables used for MaxEnt model prediction.

Code	Environmental variables	Units
Bio1	Annual Mean Temperature	℃
**Bio2**	**Mean Diurnal Range**	℃
Bio3	Isothermally (BIO2/BIO7) (* 100)	%
**Bio4**	Temperature Seasonality (standard deviation *100)	%
Bio5	Maximum Temperature of Warmest Month	℃
Bio6	Minimum Temperature of Coldest Month	℃
**Bio7**	**Temperature Annual Range (Bio5-Bio6)**	℃
Bio8	Mean Temperature of Wettest Quarter	℃
Bio9	Mean Temperature of Driest Quarter	℃
Bio10	Mean Temperature of Warmest Quarter	℃
**Bio11**	**Mean Temperature of Coldest Quarter**	℃
**Bio12**	**Annual Precipitation**	**mm**
Bio13	Precipitation of Wettest Period	mm
**Bio14**	**Precipitation of Driest Period**	**mm**
Bio15	Precipitation Seasonality (coefficient of variation)	%
Bio16	Precipitation of Wettest Quarter	mm
Bio17	Precipitation of Driest Quarter	mm
Bio18	Precipitation of Warmest Quarter	mm
Bio19	Precipitation of Coldest Quarter	mm

**Note**: * Bold text indicates the bioclimatic variables used for model construction after screening.

**Table 2.** Potential suitable area of *Parnassiawightiana* in different periods.

Period	Prediction areas (×10^4^ km^2^)			
	Low suitable area	Medium suitable area	High suitable area	Total
LIG	53.10	22.55	3.52	79.17
LGM	53.47	24.43	4.18	82.08
MH	52.21	21.71	3.45	77.37
Current	55.56	25.96	3.52	85.04
Future	54.38	24.99	3.52	82.89

**Table 3.** Potential suitable area of *Parnassiawightiana* in different periods.

Major climatic factors	Contribution rate (%)				
	LIG	LGM	MH	Current	Future
Bio12	37.2	39.8	36.3	37.9	39.2
Bio7	28	20.9	29.7	29.8	28.6
Bio10	19	17.8	19.2	17.2	18.8
Bio4	8.1	12.2	5.6	8.2	6.6
Bio2	6.4	7.2	7.6	5.3	5.5
Bio3	1.2	2.2	1.5	1.5	1.4
